# Supplementary material for: Analysis of Exosomal Cargo Provides Accurate Clinical, Histologic and Mutational Information in Non-Small Cell Lung Cancer
Source: Cancers (Basel). 2022 Jun 30;14(13):3216. doi: 10.3390/cancers14133216 (PMC9264915; doi:10.3390/cancers14133216)
Supplement: Supplementary file 1 [file cancers-14-03216-s001.zip › cancers-1747956-supplementary.pdf]

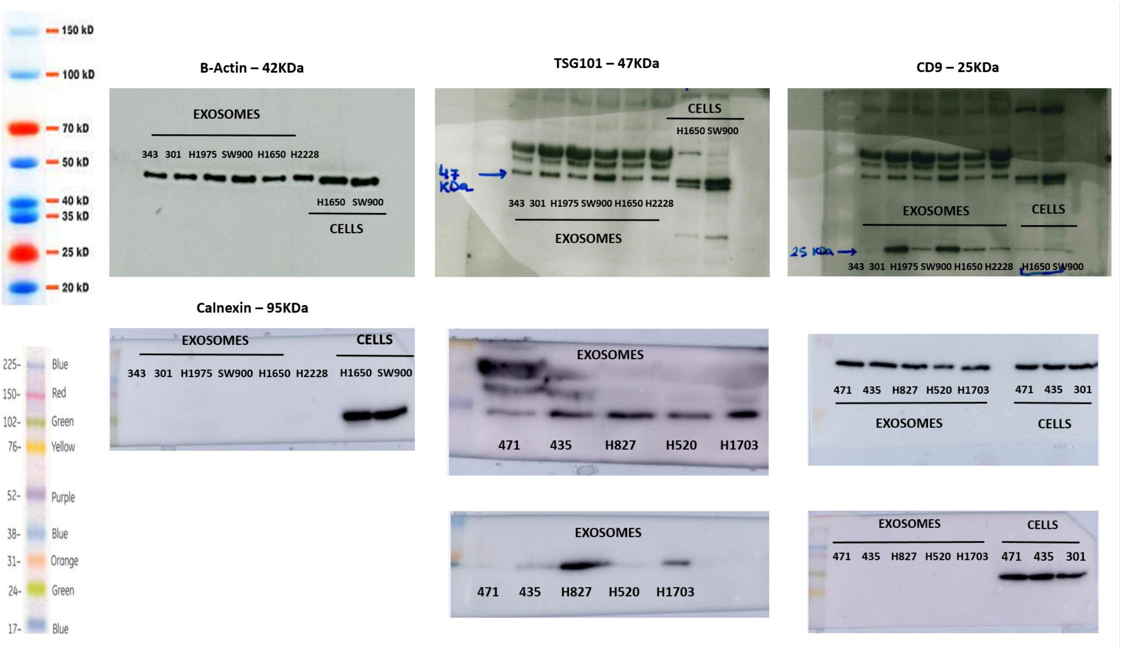

Figure S1. Original and complete immunoblots for B-actin, TSG101, CD9 and Calnexin.

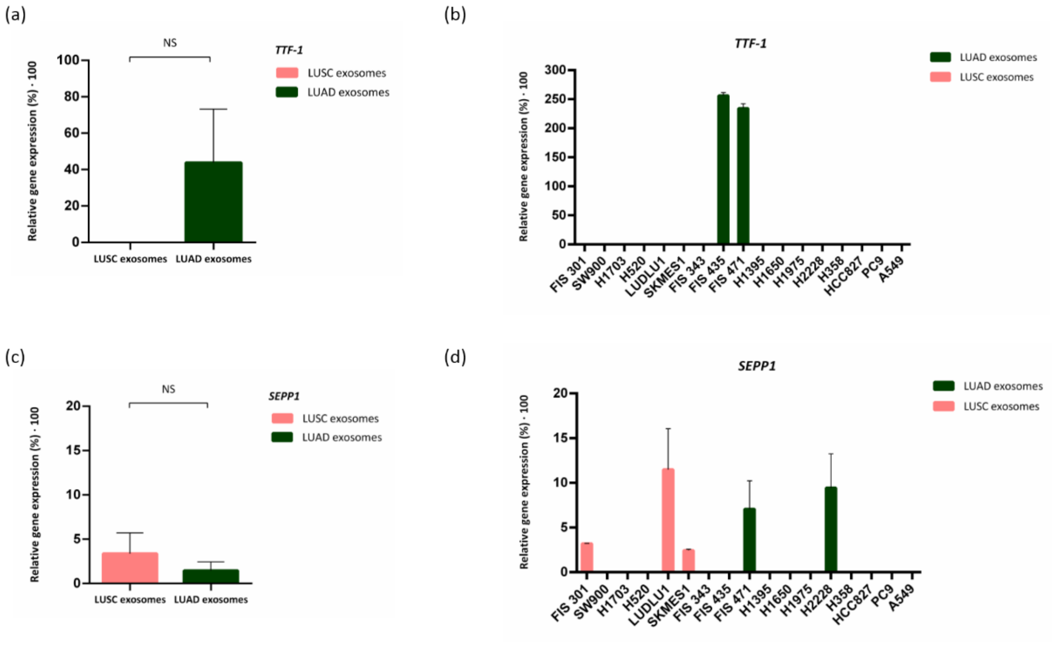

Figure S2. Analysis of *TTF-1 and SEPP1* expression in tumor-derived exosomes from 2D cell cultures. (**a-c**) Median of relative gene expression of *TTF-1 and SEPP1* measured by RTqPCR in both histological groups. Error bars represent the standard error of the mean (SEM). Dark green bars correspond to LUAD-derived exosomes while salmon bars correspond to exosomes derived from LUSC samples. (**b-d**) Mean with SD (standard deviation) of de relative gene expression of *TTF-1 and SEPP1* to reference genes *ACTB* and *GAPDH* analysed in the complete group of cell cultures-derived exosomes. NS: non-significant.

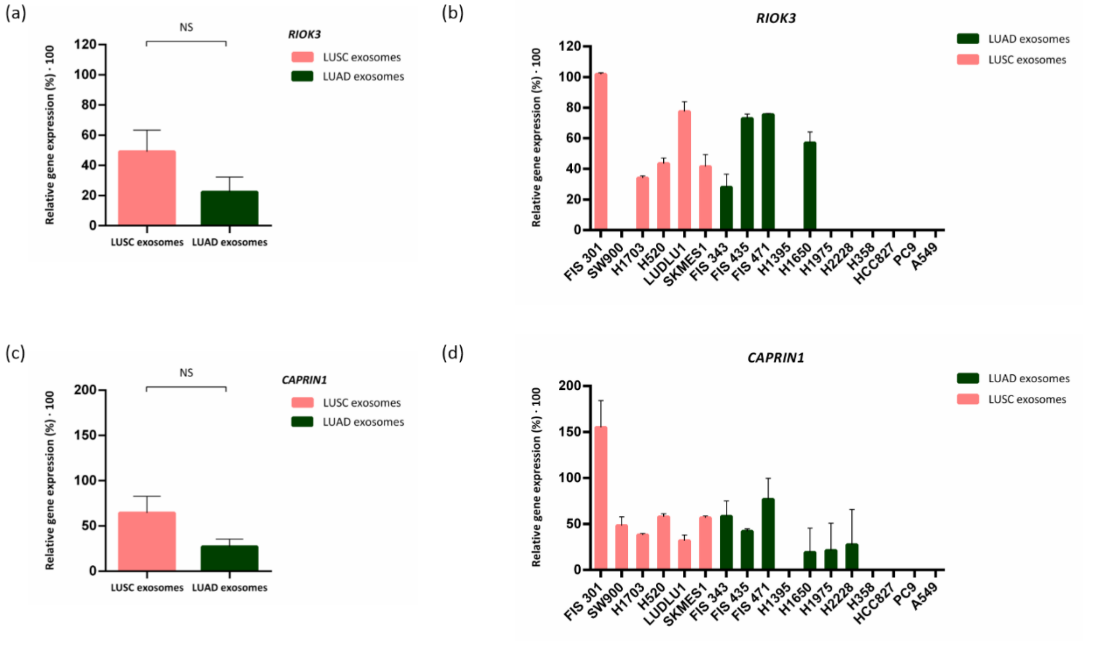

Figure S3. Analysis of *RIOK3 and CAPRIN1* expression in tumor-derived exosomes from 2D cell cultures**.** (**a-c**) Median of relative gene expression of *RIOK3 and CAPRIN1* measured by RTqPCR in both histological groups. Error bars represent the standard error of the mean (SEM). Dark green bars correspond to LUAD-derived exosomes while salmon bars correspond to exosomes derived from LUSC samples. (**b-d**) Mean with SD of de relative gene expression of *RIOK3 and CAPRIN1* to reference genes *ACTB* and *GAPDH* analysed in the complete group of cell cultures-derived exosomes. NS: non-significant.

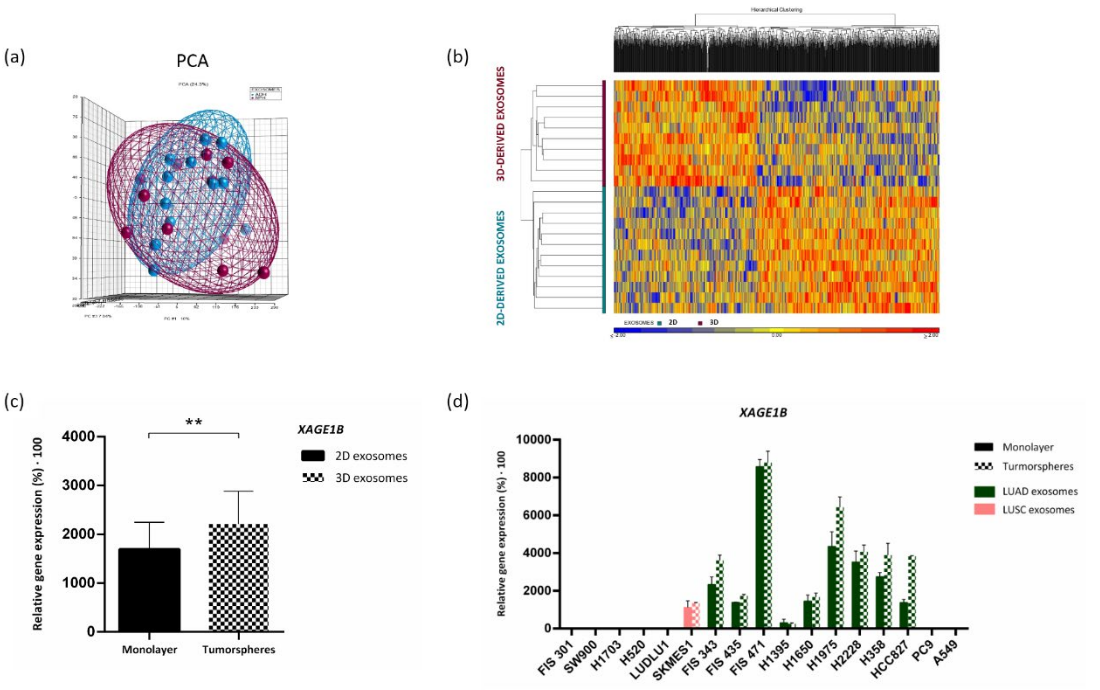

Figure S4. Validation of *XAGE1B* expression in cell cultures-derived exosomes from both models (2D-monolayer and 3D-tumorspheres). (**a**) PCA plot of exosomes samples distribution according to cell growth

models. (**b**) Hierarchical cluster analysis of differentially expressed probes between 2D and 3D groups of exosomes. Red colour represents overexpression and blue represents underexpression. Rows correspond to the exosome samples analysed while the columns represent the probes detected throughout the samples. (**c**) Median of relative gene expression of *XAGE1B* measured by RT-qPCR in both growth models of exosomes. Error bars represent the standard error of the mean (SEM). Dark green bars correspond to LUAD-derived exosomes while orange bars correspond to exosomes derived from LUSC samples. (**d**) Mean with SD of de relative gene expression of *XAGE1B* to reference genes *ACTB* and *GAPDH* analysed in the complete group of cell cultures comparing to 2D vs 3D-derived exosomes. Significance value was **p≤0.01. .

Table S1. Clinicopathological characteristics of the patients employed for the primary cell cultures establishment.

Patient code (FIS)	Gender	Age (years)	TNM stage	Histology	Smoking status	Progression & exitus	DFS (months)	Tumor Mutational status
**301**	Male	71	IIB (T3N0M0)	LUSC	Former	No	75.50	TP53 p.S261V*fs84, PIK3CA p.G118D
**343**	Female	60	IB (T2aN0M0)	LUAD	Former	Yes	7	TP53 p.R158L
**435**	Male	70	IIB (T3N0M0)	LUAD	Current	No	24	KRAS p.G12C, PIK3CA p.H1047R
**471**	Female	83	IIA (T2BN0M0)	LUAD	Never	No	27	PIK3CA p.D538N

DFS disease-free survival, LUAD lung adenocarcinoma, LUSC lung squamous cell carcinoma, WT wild type.

Table S2. Main characteristics of the cell lines included in the study.

Cell Line	Gender	Age (years)	Histology	Relevant mutations
**NCI-H1650**	Male	27	LUAD	EGFR p.E746_A750del
**NCI-H1975**	Female	UK	LUAD	EGFR p.L858R+ p.T790M<br>PIK3CA p.G118D<br>TP53 p.R273H
**NCI-H2228**	Female	UK	LUAD	*EML4-ALK fusion*,<br>TP53 p.Q331*,<br>RB1 p.E204fs*10
**NCI-H358**	Male	UK	LUAD	KRAS p.G12C
**A549**	Male	58	LUAD	KRAS p.G12S
**HCC-827**	Female	39	LUAD	EGFR p. E746_A750del<br>TP53 p.V218del
**NCI-H1395**	Female	55	LUAD	BRAF p.G469A
**PC-9**	Male	UK	LUAD	EGFR p.E746_A750del<br>TP53 p.R248Q
**SW900**	Male	53	LUSC	KRAS p.G12V<br>TP53 p.Q167*
**NCI-H520**	Male	UK	LUSC	TP53 p.W146*
**NCI-H1703**	Male	54	LUSC	-
**SKMES-1**	Male	65	LUSC	TP53 p.E298*
**LUDLU-1**	Male	72	LUSC	TP53 p.R248W

LUAD: lung adenocarcinoma, LUSC: lung squamous cell carcinoma, UK: unknown.

Table S3. List of antibodies used for immunoblot (IB), immunofluorescence (IF) and flow cytometry (FC) analyses.

Antibody	Dilution	Catalog nº	Supplier	Technique
B-Actin Anti-Mouse mAb (Clone AC-15)	1:10.000	A5441	Sigma-Aldrich	IB
Calnexin Anti-Rabbit pAb	1:1000	Ab75801	Abcam	IB
CD9 Anti-Rabbit mAb (Clone EPR2949)	1:500	Ab92726	Abcam	IB
TSG101 Anti-Mouse mAb (Clone 4A10)	1:200	Ab83	Abcam	IB
Anti-Mouse IgG (whole molecule)-Peroxidase	1:2000	A9044	Sigma-Aldrich	IB
Anti-Rabbit IgG (whole molecule)-Peroxidase	1:2000	Sc-2313	Santa Cruz Biotec.	IB
XAGE1 Anti-Goat pAb	1:100	Ab27477	Abcam	IF
CABYR Anti-Rabbit pAb	1:100	Ab243417	Abcam	IF
Alexa Fluor 555 Anti-Rabbit IgG (H+L)	1:1000	A-31572	Thermofisher	IF
Alexa Fluor 488 Anti-Goat IgG (H+L)	1:1000	A-11078	Thermofisher	IF
CD63-APC (Clone REA1055)	01:50	130-118-151	Miltenyi Biotec.	FC
CD81-PE (Clone REA513)	01:50	130-118-481	Miltenyi Biotec.	FC

Table S4. TaqMan® Gene Expression Assays used in gene expression analyses.

Gene Symbol	Gene name	Assay ID
**ACTB**≠	Actin, Beta	Hs99999903_m1
**GAPDH**≠	Glyceraldehyde-3-phosphate dehydrogenase	Hs99999905_m1
**GUSB**≠	Glucuronidase, beta	Hs01558067_m1
**CDKN1B**≠	Cyclin-dependent kinase inhibitor 1B	Hs00153277_m1
**XAGE1B/E**	X Antigen Family Member 1B/E	Hs00220764_m1
**CABYR**	Calcium Binding Tyrosine Phosphorylation Regulated	Hs00201830_m1
**NKX2-1**	NK2 Homeobox 1	Hs00968940_m1
**SEPP1**	Selenoprotein P, plasma, 1	Hs01032845_m1
**CAPRIN1**	Cell Cycle Associated Protein 1	Hs00195416_m1
**RIOK3**	RIO Kinase 3	Hs01566923_m1

≠ reference gene.

Table S5. Molecular alterations detected in cell cultures-derived exosomes with different histologies.

SW900	H1975	H358	PC9	H1650	A549	HCC827	H2228	FIS 435
LUSC	LUAD	LUAD	LUAD	LUAD	LUAD	LUAD	LUAD	LUAD
KRAS p.G12V	EGFR p.L858R/+pT790M	KRAS p.G12S	EGFR p.E746_A750del	EGFR p.E746_A750del	KRAS p.G12S	EGFR p.E746_A750 del	EML4-ALK fusion	KRAS p.G12C

LUSC: squamous cell lung cancer; LUADC: adenocarcinoma cell lung cancer.

Table S6. Enrichment analysis of pathological processes and its associated pathways based on the number of DEGs overlapped.

Process name	Hallmarks of Cancer	*p*-value	JSI	Overlap
**G0/G1 Cell Cycle Phase Transition Activation in Cancer**	Sustaining Proliferative Signaling	1.30×10^{5}	0.007	10
**TGFB Signaling activation by Blocking of Tumor Suppressors**	Evading Growth Suppressors	3.85×10^{5}	0.008	11
**Treg Cells Promote Immunosuppression in Cancer Immune Escape**	Evading Immune Destruction	7.25×10^{5}	0.006	9
**TGFB Family in Epithelial to Mesenchymal Transition in Cancer**	Activating Invasion and Metastasis	1.57×10^{4}	0.007	11
**VEGF Independent Angiogenesis in Cancer**	Inducing Angiogenesis	2.43×10^{4}	0.006	8
**ANGPT/TEK Stimulates Endothelial Cell Migration in Cancer**	Inducing Angiogenesis	3.49×10^{4}	0.005	7
**Vascular Smooth Muscle Cell/Pericyte Migration and Proliferation**	Inducing Angiogenesis	2.16×10^{3}	0.005	8
**CDH2 Activation Promotes Cancer Cell Migration and Survival**	Activating Invasion and Metastasis	2.27×10^{3}	0.005	8
**Glioma Invasion Signaling**	Activating Invasion and Metastasis	4.97×10^{3}	0.005	8

**ROS in Triggering Vascular Inflammation**	*Toxicity Induced Pathways	$7.02x10^{3}$	0.005	8
**IDO1 in Cancer Immune Escape**	Evading Immune Destruction	0.01	0.005	7
**Integrins in Cancer Cell Motility, Invasion and Survival**	Activating Invasion and Metastasis	0.02	0.006	9
**Acetylases Inhibition in Histone Deacetylation in Cancer**	Genome Instability	0.03	0.003	4

*Toxicity Induced Pathways is not a Hallmark of Cancer; JSI: Jaccard similarity index.
